# Is there still a place for etomidate in the management of Cushing’s syndrome? The experience of a single center of low-dose etomidate and combined etomidate-osilodrostat treatment in severe hypercortisolemia

**DOI:** 10.1007/s12020-024-04135-1

**Published:** 2024-12-18

**Authors:** Lukasz Dzialach, Joanna Sobolewska, Wioleta Respondek, Agnieszka Wojciechowska-Luzniak, Pawel Kuca, Przemysław Witek

**Affiliations:** 1 Department of Internal Medicine, Endocrinology and Diabetes, Medical University of Warsaw, Warsaw, Poland; 2 Department of Internal Medicine, Endocrinology and Diabetes, Mazovian Brodnowski Hospital, Warsaw, Poland

**Keywords:** Cushing’s syndrome, Ectopic ACTH syndrome, Etomidate, Osilodrostat, Severe hypercortisolaemia

## Abstract

**Purpose:**

Severe Cushing’s syndrome (SCS) is a life-threatening endocrine condition that requires prompt medical intervention. Intravenous etomidate infusion is considered to be the most effective in rapid cortisol overproduction inhibition. This single-center retrospective study aimed to present the safety and effectiveness of intravenous, low-dose, lipid-formulated etomidate infusion in patients with SCS.

**Methods:**

Seven patients with complicated SCS related to ectopic ACTH syndrome (n = 6) or Cushing’s disease (n = 1) who received low-dose etomidate infusion as a part of their cortisol-lowering treatment between April 2019 and April 2024 in the Department of Internal Medicine, Endocrinology and Diabetes of Medical University of Warsaw were included in the study. A continuous etomidate infusion was initiated at 0.01–0.02 mg/kg/h.

**Results:**

In all patients, rapid control of hypercortisolemia was achieved with a median time of 30 h (range: 12–48 h). Median serum cortisol concentration reduced from 101.9 μg/dL (range: 78.2–119.6 μg/dL) before etomidate to 19.5 μg/dL (range: 18.3–22.5) after 72 h of etomidate treatment. Etomidate infusion was followed by etomidate and osilodrostat combined treatment and then osilodrostat monotherapy in four patients; one patient underwent adrenalectomy, and two patients died during etomidate infusion due to complications of advanced malignancy.

**Conclusions:**

This study shows that low-dose and short-term lipid formulation etomidate therapy is highly effective in severe hypercortisolemia management. Combined therapy with etomidate and osilodrostat is well tolerated and could serve as a bridge in long-term SCS treatment.

## Introduction

Endogenous Cushing’s syndrome (CS) is an endocrine condition caused by cortisol overproduction, with an incidence of 2–8 cases per million people per year [1]. Most cases (approximately 80%) are caused by adrenocorticotropin (ACTH) hypersecretion—either by pituitary (Cushing’s disease [CD], 85–90%) or neuroendocrine non-pituitary tumors (ectopic ACTH syndrome [EAS], 10–15%) [2–4]. Severe CS (SCS) is a life-threatening endocrine emergency with numerous complications and high mortality, especially if it is not diagnosed promptly enough and appropriate treatment is not implemented [5]. Although SCS seems to constitute less than 5% of CS cases, it remains a great therapeutic challenge for endocrinologists and appears to be an important interdisciplinary clinical issue [6]. SCS is defined as dramatically elevated random serum cortisol concentration (>41 μg/dL; 1100 nmol/L), a 24-h urinary free cortisol greater than five-fold the upper limit of normal, and/or profound hypokalemia (<3.0 mmol/L) with the co-occurrence of at least one of the following hypercortisolemia complications: sepsis, opportunistic infection, refractory hypokalemia, uncontrolled hypertension, heart failure, gastrointestinal hemorrhage, acute psychosis, progressive debilitating myopathy, thromboembolism or uncontrolled hyperglycemia and ketoacidosis [5].

SCS requires rapid medical intervention, including symptomatic treatment of cortisol-induced complications and hypercortisolemia control with adrenal steroidogenesis inhibitors and simultaneously complex diagnostic workup determining the primary source of the disease [5, 7]. Choosing a particular steroidogenesis inhibitor is highly individualized and depends on the center’s experience, as there are no strict recommendations and therapeutic protocol for SCS.

The most effective in rapid cortisol overproduction inhibition is considered to be etomidate—a short-acting intravenous anesthetic agent [8, 9]. It reversibly inhibits 11-β-hydroxylase enzyme (CYP11B1)—the last step of cortisol biosynthesis, 17α-hydroxylase/17,20-lyase (CYP17A1) and cholesterol side-chain cleavage enzyme (CYP11A1) [9, 10]. Etomidate can be used to control hypercortisolemia quickly and as a bridging therapy to other medical or surgical interventions when the patient’s condition improves. However, despite its high therapeutic potential in SCS, it is rarely utilized in endocrinological practice.

In this article, we present the effectiveness and safety of etomidate treatment in patients with SCS hospitalized in the Department of Internal Medicine, Endocrinology and Diabetes of the Medical University of Warsaw, a tertiary center in Poland, between April 2019 and April 2024.

## Material and methods

This descriptive, retrospective study was conducted on seven patients with SCS hospitalized in the Department of Internal Medicine, Endocrinology, and Diabetes, Medical University of Warsaw, Poland between April 2019 and April 2024. The patients were included in the study if they met the criteria for SCS and received etomidate infusion as a part of their cortisol-lowering treatment.

Etomidate therapy was conducted in the conditions of an intensive internist supervision room (non-intensive care unit, ICU) with metabolic, hemodynamic, respiratory, and neurologic monitoring. Level of sedation was assessed by using the Richmond Agitation-Sedation Scale (RASS) score. The target RASS score was 0, which correlates with alert and calm patient. We used etomidate compounded in a lipid emulsion formulation (*Etomidate lipuro*) in a 2 mg/mL concentration. A continuous etomidate infusion was initiated at a rate of 0.01 to 0.02 mg/kg/h and administered intravenously through a drug pump. No initial intravenous bolus of etomidate was administered prior to infusion.

During the etomidate infusion serum cortisol concentration and potassium were assessed initially every 6 h and then every 12 h after hypercortisolemia stabilization. Blood samples were collected via an arterial cannulation. All measurements of cortisol were performed with the Abbott Alinity immunoassay (normal range: 3.7–19.7 μg/dL). Plasma ACTH was measured by using the LIAISON XL chemiluminescent immunoassay (normal range: 4,7–48,8 pg/mL).

Vital signs (heart rate, blood pressure, respiratory rate, blood oxygen saturation) assessment was conducted every 3 h. The etomidate infusion was titrated in increments of 0.005 to 0.01 mg/kg/h based of the absolute values and the rate of change in serum cortisol concentration. The infusion rate was not up titrated more frequently than every 6 h. The initial therapeutic serum cortisol concentration target was 15–25 μg/dL. This was higher than the average physiological cortisol level, but it was necessary to ensure appropriate cortisol concentration in patients with severe general condition. For patients with a milder general condition or for those whose initial severe condition improved, a therapeutic cortisol target of 10–20 μg/dL was considered appropriate. A change in the patient’s general condition (deterioration or improvement) was followed by an adequate change in the target serum cortisol. Once the target cortisol concentration was achieved, the etomidate infusion was continued to maintain serum cortisol in the desired range until implementation of long-term oral medical or surgical treatment could be initiated. The study was approved by the Ethics Committee of Medical University of Warsaw (permission number: AKBE/170/2024).

## Results

We identified 7 patients (four women) with median age 74 years (range: 31–84) treated with etomidate for SCS between April 2019 and April 2024. In six patients CS was secondary to EAS, and one patient presented with CD. Median baseline serum cortisol was 101.9 μg/dL (range: 78.2–119.6 μg/dL) and median ACTH was 603.9 pg/mL (range: 149.7–1088.0 pg/mL). Mean baseline serum potassium concentration was 2.10 ± 0.17 mmol/mL. Hypercortisolemia complications from the most common included: hypertension, hypokalemia, proximal myopathy (all patients), diabetes (six patients), sepsis (four patients), heart failure (three patients) and psychosis (two patients). All patients with EAS had distant metastases at the time of SCS diagnosis. Details of each patient are summarized in Table 1.

**Table 1:** Summary of baseline patients characteristics

Patient	Sex and age	Diagnosis	Hypercortisolemia complications	Baseline serum cortisol, μg/dL	Baseline ACTH, pg/mL	Baseline potassium, mmol/L
1	F/31	EAS (thymic LCNEC)	DM, HK, HT, P, PM	78.2	936.7	2.2
2	F/34	CD	DM, HK, HT, P, PM	106.0	167.0	1.9
3	F/61	EAS (SCLC)	DM, HF, HK, HT, PM	101.9	603.9	1.9
4	M/74	EAS (pulmonary LCNEC)	HF, HK, HT, PM, S	115.3	1088.0	2.4
5	M/75	EAS (SCLC)	DM, HF, HK, HT, PM, S	83.2	932.1	2.1
6	M/84	EAS (urinary bladder SCNEC)	DM, HF, HK, HT, PM, S	119.6	149.7	2.2
7	F/74	EAS (SCLC)	DM, HK, HT, PM, S	93.0	284.5	2.0

*ACTH* adrenocorticotropin, *CD* Cushing’s disease, *DM* diabetes mellitus, *F* female, *HF* heart failure, *HK* hypokalaemia, *HT* hypertension, *LCNEC* large-cell neuroendocrine carcinoma, *M* male, *P* psychosis, *PM* proximal myopathy, *S* sepsis, *SCLC* small cell lung cancer, *SCNEC* small cell neuroendocrine carcinoma

### Dosing and effectiveness

A continuous etomidate infusion was initiated at rate of 0.01–0.02 mg/kg/h. In six patients etomidate was a first-line therapy, and in one patient (with CD) it was introduced as an additional form of treatment due to the ineffectiveness of oral steroidogenesis inhibitor. The initial goal serum cortisol was 10–20 μg/dL in four and 15–25 μg/dL in three patients. Target serum cortisol concentration was achieved in all patients with median time of 30 h (range: 12–48 h). Median serum cortisol concentration reduced from 101.9 μg/dL (range: 78.2–119.6 μg/dL) before etomidate to 27.2 μg/dL (range: 11.3–33.4), 21.3 μg/dL (range: 18.6–24.4), and 19.5 μg/dL (range: 18.3–22.5) after 24, 48, and 72 h of etomidate treatment, respectively. The median maximum achieved etomidate infusion rate to maintain cortisol in the targeted level was 0.03 mg/kg/h (range 0.02–0.08 mg/kg/h). The infusion was continued for a median duration of 14 days (range: 5–19 days). Normokalaemia was achieved with median time of 18 h (range: 6–72 h). Hypokalemia was corrected in all patients through combined intravenous and oral administration of potassium along with spironolactone 50–200 mg/day per os in five and potassium canrenoate 200-400 mg/day intravenously in two patients. Mean serum potassium concentration rise from 2.10 ± 0.17 mmol/mL before etomidate to 3.40 ± 0.86 mmol/L, 4.10 ± 0.67 mmol/L, and 4.20 ± 0.48 mmol/L after 24, 48, and 72 h of etomidate treatment, respectively. Glucose control improved from the beginning and in three out of six patients with cortisol-induced diabetes it was possible to withdrawn insulin therapy during etomidate infusion. Similarly, improvement in hypertension control was observed in all patients, which allowed for reduction (in four patients) or discontinuation (in two patients) antihypertensive therapy during etomidate infusion. Data regarding the treatment with etomidate are summarized in Table 2 and Table 3.

**Table 2:** Summary of etomidate treatment in presented patients

Patient	Starting etomidate dose, mg/kg/h	Max etomidate dose, mg/kg/h	Target cortisol, μg/dL	Time totarget cortisol, hours	Time to potassium normalization, hours	Duration of etomidate infusion, days	Reason to stop etomidate therapy
1	0.02	0.06	10–20	36	24	11	Adrenalectomy
2	0.015	0.03	10–20	48	72	19	Osilodrostat implementation
3	0.02	0.08	10–20	12	6	19	Osilodrostat implementation
4	0.01	0.015	15–25	30	12	5	Death
5	0.015	0.025	15–25	30	36	17	Osilodrostat implementation
6	0.015	0.06	15–25	18	12	11	Death
7	0.01	0.025	15–25	24	18	14	Osilodrostat implementation

**Table 3:** Serum cortisol response to etomidate therapy during first 72 h of treatment

**Time of etomidate treatment, hours**	0	12	24	36	48	60	72
Serum cortisol, μg/dL	101.9 (78.2–119.6)	41.0 (20.4–55.8)	27.2 (11.3–33.4)	24.4 (16.7–36.5)	21.3 (18.6–24.4)	21.2 (12.4–24.5)	19.5 (18.3–22.5)
Etomidate dose, mg/kg/h	0.015 (0.01–0.02)	0.02 (0.01–0.02)	0.02 (0.01–0.03)	0.02 (0.01–0.03)	0.02 (0.02–0.03)	0.02 (0.02–0.04)	0.025 (0.02–0.04)

Values are shown as medians and ranges

**Fig. 1:**
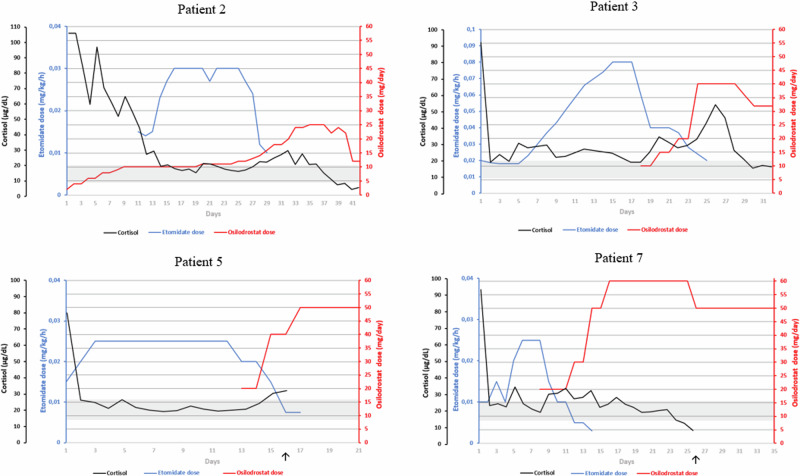
The evolution of serum cortisol levels during treatment with etomidate and osilodrostat is depicted. Cortisol values represent the average of 2 to 4 measurements taken during the day. The shaded area indicates the target cortisol level. An arrow marks the beginning of hydrocortisone administration for patients who initiated the “block and replace” treatment regimen with osilodrostat

In four patients, the etomidate infusion was continued until the oral steroidogenesis inhibitor (osilodrostat) was successfully implemented, and in one, until the adrenalectomy. Before switching to osilodrostat monotherapy, patients were receiving combination therapy (etomidate and osilodrostat). The median duration of combined etomidate and osilodrostat therapy was 7 days (range: 5–19 days). Two patients continued osilodrostat in „block and replace” approach and in two patients osilodrostat was titrated by gradual dose increase. Data regarding the treatment with osilodrostat are summarized in Table 4 and. Figure 1 presents evolution of serum cortisol concentration during combined treatment with etomidate and osilodrostat.

**Table 4:** Summary of osilodrostat treatment in presented patients

Patient	Time of combined therapy, days	Osilodrostat treatment strategy	Max osilodrostat dose, mg/d	Maintenance osilodrostat dose, mg/d
2	19	Titration	25	12
3	8	Titration	40	32
5	4	Block and replace	50	50
7	7	Block and replace	60	50

### Safety

All patients tolerated their etomidate infusion well. At baseline, five of them had a RASS score of 0. Two patients initially presented with hypercortisolemia-induced psychosis, one with a RASS score of +1 and second with +2, that improved to 0 during etomidate treatment. One patient with an initial 0 RASS score experienced slight somnolence with a RASS score of -1. In the rest of the patients the initial RASS score remained unchanged throughout the etomidate infusion. Two patients died during the etomidate infusion: one due to antibiotic resistant *Acinetobacter baumannii* sepsis and the other from respiratory failure secondary to unresectable large cell neuroendocrine carcinoma of the lung. One patient experienced adrenal insufficiency episode with hypotension requiring transient hydrocortisone implementation and etomidate dose reduction. No substantial changes in renal function during the etomidate infusion were observed. Mild hyperkaliemia occurred in one patient, but reducing potassium supplementation was sufficient to correct it.

## Discussion

In this study, we report the effectiveness and safety of continuous intravenous low-dose, lipid-formulated etomidate infusion treatment outside the ICU in seven patients with SCS in our center’s experience. All analyzed patients achieved a target cortisol level and normalized serum potassium concentration within a median of 30 and 18 h, respectively. The etomidate infusion served as bridging therapy for osilodrostat monotherapy in four patients and for adrenalectomy in one patient.

SCS is a challenging and life-threatening condition accompanied by acute cardiovascular, infectious and metabolic complications, leading to high mortality. Rapid control of cortisol levels is essential with simultaneous preventive and curative treatments of cortisol-induced comorbidities. In most cases, SCS is associated with EAS—neuroendocrine tumors found in different locations, with varying degrees of histological differentiation and aggressiveness, may secrete ACTH and lead to SCS [7, 11]. Nevertheless, SCS may also occur in patients with CD and ACTH-independent hypercortisolemia.

Etomidate is an imidazole derivative, intravenous anesthetic agent, and one of its side effects includes suppression of adrenocortical function [12, 13]. It reversibly inhibits CYP11B1, CYP17A1 and (at higher doses) CYP11A1, ultimately inhibiting cortisol biosynthesis [13]. Inhibition of adrenal steroid synthesis significantly limits the use of etomidate in anesthetic practice but gives the possibility of its off-label use in patients with SCS. Etomidate allows for highly effective and rapid control of hypercortisolemia, and normalization of cortisol concentration could be achieved within a dozen or so hours [8, 9]. In addition, it is the only steroidogenesis inhibitor which may be administered parenterally, therefore, it can also be used as a first-line treatment in severely ill patients or when oral cortisol-lowering agents cannot be used [5, 9, 10].

Since the first report of etomidate-induced steroidogenesis inhibition [13], several studies of its effectiveness in hypercortisolemia control have been published, and various therapeutic protocols have been proposed [8, 14–17]. A standard etomidate infusion protocol requires patient hospitalization in the ICU. Initially, the patient receives a bolus etomidate dose of 2.5–5 mg over 2–3 min, followed by a continuous infusion (0.1–1.0 mg/kg/h), titrated according to the therapeutic response. However, such a procedure results in a complete blockade of adrenal steroid biosynthesis and necessitates implementing hydrocortisone substitution [8, 9, 14]. It also increases the risk of adrenal crisis and propylene glycol toxicity if etomidate in propylene glycol formulation is used. Therefore, the possibility of using low-dose etomidate treatment to control SCS was suggested, which does not require hospitalization of the patient in the ICU [16]. Constantinescu et al. studied the outcome of two series of SCS patients treated either with a standard etomidate dose in ICU or a low dose in non-ICU settings. The low-dose group achieved target cortisol levels slower, but without inducing adrenal insufficiency or other side effects, and without the need for intensive care resources [14]. In addition to this data, Carroll et al. proposed a standardized intravenous low-dose etomidate infusion protocol [15].

In our cohort of patients, we used an even lower starting dose of etomidate (0.01–0.02 mg/kg/h) and omitted its initial loading dose. In all patients, etomidate effectively reduced cortisol concentration to the target range in a median time of 30 h. That further confirms the use of a very low dose of etomidate in SCS management. Interestingly, the median time to reach the target cortisol concentration in our group was shorter, despite using lower doses of etomidate compared to the studies mentioned [14, 15]. This could be attributed to the higher target cortisol concentration in some of the septic patients we analyzed and the older age of the group we studied; elderly patients require decreased etomidate doses due to reduced protein binding and clearance [18]. Indeed, the longest time to achieve the target cortisol concentration was observed in the youngest patients in our group. Therefore, doses of etomidate need to be individualized in each clinical situation and rely on patient’s clinical careful assessment. The standard treatment protocol should be rather considered only in the most severe, critical cases requiring hospitalization in the ICU. It is also important to note that the effect of etomidate on adrenal blockage may continue even after treatment discontinuation due to accumulation in the subcutaneous tissue [9].

How to qualify patients with SCS for treatment in or outside the ICU remains to be determined. It was previously recommended that etomidate treatment in patients with SCS only occur in the ICU setting [8]. However, this was mainly related to using a classic therapeutic protocol and high doses of etomidate. In previous years, collected data indicate that low-dose etomidate infusions can be administered outside of the ICU and do not necessarily have to be conducted under anesthesiologic control. In our opinion, the decision regarding the place of treatment should be made primarily based on the patient’s clinical condition and the given center’s experience in managing patients in severe state. In the analyzed group, endocrinologists carried out the treatment within the intensive internist supervision room, a coherent part of our department intended to treat patients in general severe condition. This room has a system of continuous patient monitoring, so its concept is similar to the ICU. Most internal medicine departments in Poland have at least one bed adapted for intensive supervision. In case of any doubts regarding etomidate pharmacotherapy, we could consult the ICU team. Similarly, in case of a sudden deterioration of the general condition, the patient could be qualified for further treatment in the ICU. However, we are aware that in different countries and healthcare systems, the organization of given units varies and the treatment conditions of specific groups of patients may differ significantly. Not all endocrinology centers have the expertize and appropriate facilities to allow treatment to be carried out in a general ward. On the other hand, intensive care specialists might not be familiar with the complexity of SCS and the specifics of therapy for such patients. In such cases, etomidate therapy should be conducted in close cooperation between the endocrinologist, the intensive care specialist, and the anesthesiologist. Some authors suggest that etomidate treatment could be initiated in the ICU and continued in the general ward after the patient has clinically stabilized [19]. However, given the rarity and potentially lethal complications of SCS, we strongly recommend that these patients be managed in tertiary centers with experience in fully managing this condition.

It is considered that formulations of etomidate in a lipid emulsion should be preferred due to possible propylene glycol toxicity [9, 15], which may result in plasma hyperosmolarity, lactic acidosis, thrombosis, and acute kidney injury [10, 15, 20]. However, published data on its use in patients with SCS are lacking, as in the most reports, etomidate in propyl glycol formulation was used [14, 15, 17]. To our knowledge, this report summarizes the largest cohort of patients treated with lipid formulation of etomidate, confirming its equal effectiveness to the propylene glycol formulation. Considering the known and potential complications of propylene glycol toxicity, the lipid formulation of etomidate should be regarded as superior in the case of etomidate use consideration in SCS.

Regardless of the formulation, etomidate treatment appears to have a high safety profile as an cortisol-lowering agent, and its use in SCS has been reported in adolescents [21, 22], children [23], and even infants [24]. Sedation is typically observed at higher doses than those that suppress cortisol production [9, 10]. However, the level of sedation should be assessed through the etomidate treatment, for example, using the RASS score [15]. In our cohort, only one patient experienced slight somnolence with a RASS score of −1 during etomidate infusion. The same patient presented with a brief episode of adrenal insufficiency, requiring temporary hydrocortisone implementation and etomidate dose reduction. However, it occurred during the infusion of a relatively low dose of etomidate (0.015 mg/kg/h) during the second day of the treatment. This patient had significantly increased liver enzyme activity, most likely secondary to numerous liver metastases and cortisol-induced hepatotoxicity. Because hepatic enzymes metabolize etomidate, liver damage presumably led to increased etomidate half-life. Very rarely does etomidate cause significant hepatotoxicity, which, nevertheless, must be considered [18]. However, the increased activity of liver enzymes in our patient was not likely the result of etomidate therapy itself, as it was already observed at baseline before the introduction of etomidate. Although the activity of liver enzymes initially increased during treatment, it significantly improved during subsequent days of therapy. Two of the presented patients died during the etomidate infusion; however, their death was a result of their end-stage malignancy disease and initial hypercortisolemia complications, and was not etomidate-related.

We have previously published our initial experience with etomidate and osilodrostat combination therapy in a 32-year-old female with SCS due to CD [25]. At that time, evidence for the osilodrostat monotherapy in SCS and dose titration strategy was limited. We initiated the patient’s treatment with osilodrostat at a gradually increased dose. After one week, due to an insufficient response, we decided to start treatment with etomidate. However, we continued osilodrostat simultaneously. This approach aimed to control hypercortisolemia quickly and then cross-titrate the doses of osilodrostat and etomidate to eventually discontinue the etomidate infusion and maintain hypercortisolemia control with osilodrostat monotherapy. This patient is also included in the current study. Since then, real-world evidence of osilodrostat use in SCS and EAS has emerged [26, 27], and an algorithm for the practical use of osilodrostat in SCS has been proposed [27]. That raises the question of whether there is still a place for etomidate in managing SCS in the era of new oral steroidogenesis inhibitors.

Despite increasing evidence of the effectiveness of osilodrostat as a first-line therapy in SCS, its global availability, especially considering the treatment price, is limited. For example, in Poland, innovative medications are financed through a specific emergency access procedure. The process of obtaining osilodrostat for a patient takes at least several days. It may not significantly affect the prognosis in cases of milder forms of CS, but in patients with SCS, even a delay of several days in the hypercortisolemia treatment may have disastrous consequences. On the other hand, another oral steroidogenesis inhibitor, ketoconazole, is not registered for the treatment of CS in Poland and currently is unavailable in our country. Moreover, metyrapone is not reimbursed in Poland, and the hospital must cover the eventual treatment fee. Mitotane is an additional therapeutic option, but it is mainly intended for patients with adrenocortical cancer and its use in SCS is limited due to the relatively slow onset of action. Taking this into account, etomidate therapy remains the only salvation for patients with SCS, serving as bridging therapy until the possibility of treatment with osilodrostat is available. Ultimately, the etomidate infusion was followed by combined etomidate and osilodrostat treatment and, eventually, osilodrostat monotherapy in four of the presented patients. Continuous etomidate infusion was maintained while initiating osilodrostat treatment to prevent rebound hypercortisolemia. The combination treatment aimed to provide stable control of hypercortisolemia while increasing the osilodrostat dose and reducing the etomidate dose until its discontinuation. In two patients, the osilodrostat dose was gradually increased, while in the other two patients, osilodrostat was implemented at a high dose with rapid escalation and continued in the „block and replace” regimen. However, no specific protocol was followed, and therapeutic decisions were made based on the patient’s clinical condition and laboratory tests results. This significantly limits the possibility of proposing a specific scheme for transitioning from etomidate to osilodrostat. However, on Fig. 1 we present graphs showing the evolution of the mean serum cortisol concentration to illustrate the combined treatment process with etomidate and osilodrostat.

Our study has several limitations. The most important one is relatively small number of participants; however, it is still significant when taking into account how extremely rare SCS is and how infrequently etomidate is utilized in this indication. Our study also lacks patients with ACTH-independent CS. The study has a retrospective nature, therefore presents the results of real clinical practice, as no strict treatment protocol was followed.

## Conclusions

Low-dose and short-term etomidate therapy is effective in SCS control and safe to conduct in non-ICU settings. Our experience also suggests that initiating treatment with an etomidate bolus may not always be necessary, but this requires further investigation. Lipid formulation of etomidate appears to have similar effectiveness to propylene glycol-formulated one; however, given the potential propylene glycol-induced toxicity, etomidate in lipid formulation should be preferred in SCS management. In cases where quick access to a potent oral steroidogenesis inhibitor is not available, etomidate may be the only viable treatment option for SCS patients. Furthermore, combined therapy with etomidate and osilodrostat was well tolerated and could serve as a bridge in long-term SCS treatment. However, this requires further reports on the use of other oral steroidogenesis inhibitors in combination with etomidate.

## Data Availability

No datasets were generated or analysed during the current study.
